# A randomized controlled study of a psycho-behavioral intervention combined with a non-benzodiazepine to improve perioperative sleep quality in patients undergoing knee arthroplasty

**DOI:** 10.3389/fsurg.2023.1113577

**Published:** 2023-07-14

**Authors:** Wang Jing, Zhao Chunlin, Yang Xue, He Tingting, Yuan Linyan, Chen Xiao, Li Lingli

**Affiliations:** West China Hospital, Sichuan University/West China School of Nursing, Sichuan University, Chengdu, China

**Keywords:** psycho-behavioral interventions, zolpidem tartrate, knee arthroplasty, sleep quality, randomized controlled trial

## Abstract

**Objective:**

To clarify the effectiveness and safety of psycho-behavioral intervention combined with a non-benzodiazepine to improve perioperative sleep quality in patients undergoing knee arthroplasty, and also to explore the optimal dosage of non-benzodiazepine (oral zolpidem tartrate) to form a standardized sleep quality management process to promote accelerated recovery of patients.

**Methods:**

240 patients undergoing initial unilateral total knee arthroplasty between January and December 2016 were prospectively included and randomly divided into blank control group (group A), psycho-behavioral intervention group (group B), zolpidem tartrate 10 mg group (group C), and psycho-behavioral intervention + zolpidem tartrate 5 mg group (group D). Sleep, pain, psychological, and knee function scores were compared.

**Results:**

There were significant differences between the four groups with respect to Pittsburgh sleep quality index scores, sleep efficiency, Epworth Sleepiness Scale scores, inflammatory indices, pain, and enhanced recovery after surgery indices during hospitalization (*P *< 0.05).

**Conclusion:**

Psychological behavioral intervention combined with non-benzodiazepine can improve the perioperative quality of sleep. Compared with drug intervention alone, it reduces the dosage of non-benzodiazepine, alleviates anxiety, improves patient satisfaction, and enhances the quality of life.

## 1. Introduction

With the modernization of society, widespread use of modern transportation, and the global trend of population aging, the incidence of joint injuries and orthopedic trauma has shown a progressive increase worldwide (1–3). The number of patients undergoing hip and knee replacement surgery worldwide exceeds one million annually, and the number is increasing every year placing a heavy burden on health care systems (4). The development of Enhanced Recovery After Surgery (ERAS) has brought a solution to this challenge. Due to the traumatic nature of orthopedic surgery, forced postoperative positions, and significant pain, perioperative sleep quality issues are a key impediment to the patients' accelerated recovery after surgery, with a prevalence of up to 50% (5). Poor sleep quality affects immunity, increases infection rates, causes poor hospital experience, delays rehabilitation function, prolongs hospital stay, and increases hospital costs (6). Therefore, in-depth characterization of the perioperative sleep quality problems and its determinants in patients undergoing hip/knee replacement patients, and exploration of effective interventions to promote accelerated recovery of patients are key imperatives. The purpose of this study was to determine the efficacy and safety of psycho-behavioral interventions administered in combination with a non-benzodiazepine in improving perioperative sleep quality in patients undergoing knee replacement. We explored the effects of different intervention programs on sleep, inflammatory indices, and pain scores in patients undergoing knee replacement and explored the relationship between sleep, pain, and accelerated rehabilitation. Our findings may help inform a standardized protocol for sleep quality management and promote accelerated rehabilitation of patients.

## 2. Objects and methods

### 2.1. Study objects

#### 2.1.1. Sample size calculation

The sample size was estimated using the following formula:

$$N\;=\;(1\;+\;1/k)(u_{\alpha }/2+u_{\beta }{)}^{2}\sigma^{2}/\delta^{2}$$

*α *= 0.05, *β *= 0.10, *δ *= 1.4, *σ *= 2.5 were obtained by referring to the literature. u*_α_*_/2_ and u*_β_* are the u values of the first-class error and the second-class error probability, respectively (1.96 and 1.28, respectively). Based on the formula, a sample size of 45 was required for each group. Factoring a drop-out rate of 20%, the number was 54 cases, which was rounded to 60 cases in each group. Finally, 240 patients who were admitted in the orthopedic joint ward of a tertiary care hospital for undergoing unilateral knee arthroplasty between January and December 2016 were included. Inclusion criteria: (1) patients aged ≥18 years undergoing first unilateral knee arthroplasty; (2) written informed consent obtained from patients/families; (4) Pittsburgh sleep quality index (PSQI) ≤7 at admission. Exclusion criteria: (1) patients who were unwilling to cooperate; (2) patients with severe anxiety at admission. Subjects who had a change in condition and could not continue to participate in the trial were also excluded.

### 2.2. Groups

A randomized single-blind control method was used, with blinding of the patient. Patients were divided into 4 groups using a computer-generated random number sequence. Blank control group (group A): only the routine process of accelerated rehabilitation in the perioperative period of total knee arthroplasty was performed according to the routine process; psycho-behavioral intervention group (group B): centrally arranged to Ward B nursing group, using psycho-behavioral intervention of sleep education and relaxation therapy. Psycho-behavioral interventions were implemented by trained charge nurses on patients three times a day at 10:00, 16:00 and 21:00 for 20 min each time; Zolpidem Tartrate 10 mg group (group C): Zolpidem tartrate tablet 10 mg was administered 30 min before bedtime every night; psycho-behavioral intervention + zolpidem tartrate 5 mg group (Group D): centrally arranged to Ward D care group. Psycho-behavioral intervention was started on the day of admission by a uniformly trained charge nurse, three times a day for 20 min each time. 5 mg of Synthroid was administered from 2 d before to 5 d after surgery at 30 min before bedtime under the guidance of the charge nurse. Patients were randomly grouped upon admission and assigned to 4 different wards without seeing each other to avoid contamination.

### 2.3. Conventional treatment and care

All patients were operated by the same surgical team using the same perioperative procedure (6–8 h preoperative fasting, 2 h abstinence from drinking, combined intravenous inhalation anesthesia, and postoperative routine administration of cefuroxime 1,500 mg 2 times/day intravenously). If patients showed signs of infection such as pulmonary infection and incisional cellulitis, the antibiotic was changed to levofloxacin hydrochloride injection 500 mg 1 time/day intravenously. All patients were given peri-incisional ice for 24 h upon return to the ward and a sequential intravenous drip of tranexamic acid 1 g three hours after surgery. If plasma drainage is placed, the drainage tube should be removed as soon as the bleeding tends to stop (no obvious bleeding in the drainage tube or serum in the drainage tube). The drainage tube can be removed on the day of surgery or on the first postoperative day, and the drainage volume should be recorded. Analgesic medications were applied using a three-tiered principle, with primary medications started 3 d before surgery and continued postoperatively; secondary and tertiary analgesics were used as needed. Patients were instructed to passively extend and flex the knee in bed on the day of surgery for functional exercise and walk without limp using a walker. The environment in the ward was maintained quiet, with the lights dimmed at lunchtime and night, and focus on treatment and early extubation.

### 2.4. Data collection and observation indicators

1.4.1 Baseline information The table includes basic information such as age, gender, height, weight, operation time, anesthesia classification, number of rooms, operation site, diagnosis, and comorbidities. It was collected after the study subjects signed the informed consent form, and the information was mainly obtained from the medical records, nursing records and the study subjects themselves, and recorded by the uniformly trained data collectors.

1.4.2 Sleep quality evaluation index PSQI scale (Pittsburgh Sleep Quality Index) was investigated and assessed by the data collector at 15:00 on the day of patient admission and 9:00 on the day of discharge. Sleep efficiency was assessed by the data collector who instructed the patients to wear sleep monitors and exported the data at 9:00 every day to record the patients' sleep efficiency 1 d before and 1 d, 3 d and 5 d after surgery.ESS (Epworth sleep scale) scale was investigated and assessed by the data collector at 15:00 before and 1 d, 3 d and 5 d after surgery.

1.4.3 Hematological indexes were ordered by the supervising physician, and the responsible nurse drew blood on an empty stomach before and 1 d, 3 d, and 5 d after surgery in the morning and sent it immediately for testing ESR (Erythrocyte Sedimentation Rate), CRP (C-reaction protein), and IL-6 (Interleukin-6). The data collector uniformly extracts the data from the test report.

1.4.4 VAS pain scores (Visual Analogue Scale) were collected by the data collector at 15:00 each day before and 1 d, 3 d, and 5 d after surgery for walking, resting, and knee flexion, and at 20:30 for nighttime VAS scores.

1.4.5 Knee circumference The circumference of the affected knee was measured with a soft ruler at the midpoint line of the patella. The measurements were collected by the data collector with a soft ruler before and 1 d, 3 d and 5 d after surgery starting at 15:00.

1.4.6 GAD-7 score (Generalized Anxiety Disorder-7) The GAD-7 score was assessed by the data collector at 15:00 on the day of admission and at 9:00 on the day of discharge.

1.4.7 Patient satisfaction scores were collected by the data collector at 15:00 on 1 d, 3 d, and 5 d postoperatively.

1.4.8 Knee mobility, AKS score (the American Knee Society) The normal knee joint is generally flexed at 0–130°. The AKS score of the knee contains two parts: the clinical score of the knee and the functional score. The clinical score includes pain, mobility and stability; the functional score includes evaluation of walking ability and the ability to walk up and down stairs. It was completed by the data collector at 15:00 on the day of admission, and the knee mobility was retested at 15:00 from 1 d, 3 d and 5 d after surgery, and the AKS score was retested in the outpatient clinic at 3 w after discharge.

1.4.9 Quality of life SF-12 score (the 12-Item Short Form Health Survey) Completed by the data collector at 15:00 on the day of admission and retested at the outpatient clinic 3 w after discharge.

1.4.10 Perioperative Adverse Reactions A dedicated data collector will collect and record the patient's possible perioperative adverse reactions while measuring the data: difficulty concentrating, exhaustion, memory difficulties, dry mouth, sweating, headache, diarrhea, gastrointestinal reactions, rebound insomnia, early awakening, hangover effects, etc.

### 2.5. Statistical methods

SPSS 21.0 software was used for data analysis. Data are presented as mean ± standard deviation or as frequency (percentage). Between-group differences were assessed using the chi-squared, rank sum test, ANOVA or two independent samples *t* test.

### 2.6. Quality control

Strict inclusion and exclusion criteria were used to select the study subjects. Patients were divided into four different ward care groups to avoid cross-contamination. All relevant personnel underwent standardized training and the operations were consistent as much as possible, except for the interventions, to reduce confounding bias. The same calibrated measuring instruments were used throughout the trial to avoid operational interference as much as possible and to reduce bias due to confounding factors.

## 3. Results

### 3.1. Baseline information

A total of 247 patients qualified the inclusion and exclusion criteria and were enrolled; of these, 5 patients were lost to follow-up and 2 patients had missing data. Therefore, 240 patients completed the trial in the end. The baseline indicators of the study population are shown in Table 1. There were no significant differences between the four groups with respect to age, sex, height, weight, body mass index (BMI), or time to surgery at baseline (*P *> 0.05).

**Table 1:** Comparison of baseline information.

Type	A	B	C	D	*P*-value
Number of cases	60	60	60	60	
Age	68.08 ± 9.4	66.31 ± 8.3	64.9 ± 7.3	65.3 ± 7.3	0.15
Sex					0.93
Male	23	26	25	23	
Women	37	34	35	37	
BMI	25.50 ± 3.80	26.00 ± 3.70	26.50 ± 3.50	25.90 ± 3.80	0.54
Surgery time	61.15 ± 6.40	63.36 ± 5.90	62.50 ± 6.30	61.40 ± 7.10	0.27
Anesthesia grading	2.12 ± 0.58	2.25 ± 0.65	2.15 ± 0.60	2.30 ± 0.50	0.34
Patients in the ward					0.27
Single room	1	2	2	3	
Double room	7	8	6	9	
Six-person room	22	24	28	24	
Seven-person room	30	26	24	24	
Operation site					0.77
Right knee	33	28	32	33	
Left knee	27	32	28	27	
Diagnosis					0.88
Osteoarthritis	46	51	46	49	
Endorheumatoid arthritis	11	8	12	9	
Other	3	1	2	2	
Complication (medicine)					0.94
Hypertensive	5	2	5	5	
Diabetes	2	3	2	2	
Osteoporosis	4	2	2	4	
Chronic obstructive pulmonary disease	3	4	4	3	
Other	1	3	1	1	

### 3.2. Sleep

The percentage of patients with sleep quality PSQI scores >7 during hospitalization was 58.33%, 31.67%, 18.33%, and 11.67% (*P *< 0.05). Sleep efficiency and ESS scores at 1 d, 3 d, and 5 d postoperatively were better in groups B, C, and D than in the blank control group; groups C and D were significantly better than groups A and B (*P *< 0.05). Sleep efficiency at 1 d postoperatively was the lowest and ESS score was the highest (Table 2).

**Table 2:** Comparison of sleep efficiency (%) and ESS score results.

Categories	PRE-OP	POD1	POD3	POD5
**Sleep efficiency**				
A	80.89 ± 3.96	52.86 ± 7.28	64.79 ± 7.07	71.46 ± 5.12
B	81.01 ± 3.56	56.53 ± 6.40	68.66 ± 6.85	75.67 ± 4.55
C	80.48 ± 3.20	61.33 ± 6.40	72.96 ± 6.85	79.77 ± 4.55
D	81.04 ± 2.66	62.47 ± 5.28	74.07 ± 5.17	80.68 ± 3.93
*P*	0.7904	**<0** **.** **05**	**<0** **.** **05**	**<0** **.** **05**
**ESS**				
A	4.40 ± 0.89	8.20 ± 1.12	6.80 ± 1.05	5.73 ± 1.30
B	4.47 ± 0.72	7.20 ± 1.12	5.90 ± 1.05	4.83 ± 1.26
C	4.53 ± 0.89	6.00 ± 1.12	4.90 ± 1.05	4.53 ± 1.26
D	4.67 ± 0.88	5.84 ± 1.04	4.85 ± 1.03	4.27 ± 1.18
*P*	0.3573	**<0** **.** **0001**	**<0** **.** **0001**	**<0** **.** **0001**

The bold values represent the statistically significant difference in P-values (i.e., *p* ≤ 0.05).

### 3.3. Inflammatory indicators and pain scores

#### 3.3.1. Inflammatory indicators: ESR, CRP and IL-6

There were significant differences between the four groups with respect to the ESR, CRP, and IL-6 levels at 1 d, 3 d and 5 d postoperatively, respectively (*P *< 0.05). Groups B, C, and D were superior to the blank control group, and groups C and D were superior to groups A and B. The differences were statistically significant (*P *< 0.05) (Table 3).

**Table 3:** Comparison of inflammatory indicators.

Categories	PRE-OP	POD1	POD3	POD5
**ESR**				
A	26.53 ± 7.97	35.53 ± 6.96	58.00 ± 8.28	55.33 ± 9.59
B	25.53 ± 8.83	29.33 ± 10.25	53.43 ± 7.30	48.57 ± 9.21
C	25.4 ± 7.77	25.93 ± 7.62	49.07 ± 8.48	42.07 ± 7.07
D	25 ± 7.96	25.23 ± 8.47	47.25 ± 9.64	40.8 ± 8.30
*P*	>0.05	**<0** **.** **0001**	**<0** **.** **0001**	**<0** **.** **0001**
**CRP**				
A	3.69 ± 2.76	53.26 ± 11.82	91.84 ± 11.81	43.78 ± 12.07
B	3.67 ± 2.24	45.80 ± 13.61	80.49 ± 13.63	33.49 ± 8.03
C	3.63 ± 2.68	34.98 ± 8.25	75.46 ± 11.83	30.85 ± 8.30
D	3.51 ± 1.79	33.43 ± 8.83	73.69 ± 13.11	28.16 ± 12.17
*P*	0.9767	**<0** **.** **0001**	**<0** **.** **0001**	**<0** **.** **0001**
**IL-6**				
A	3.38 ± 1.48	96.44 ± 21.25	37.75 ± 12.03	21.24 ± 8.12
B	3.40 ± 1.29	78.76 ± 10.84	26.45 ± 9.53	13.83 ± 3.77
C	3.50 ± 1.39	70.02 ± 10.56	23.11 ± 5.66	9.57 ± 4.09
D	3.50 ± 1.39	71.49 ± 8.79	23.25 ± 7.22	9.83 ± 3.40
*P*	0.9404	**<0** **.** **0001**	**<0** **.** **0001**	**<0** **.** **0001**

The bold values represent the statistically significant difference in P-values (i.e., *p* ≤ 0.05).

#### 3.3.2. Pain scores: walking, resting, nocturnal, flexed knee VAS scores

Patients in the four groups showed significant differences (*P *< 0.05) with respect to postoperative walking, resting, nocturnal, and hip flexion VAS pain scores at 1 d, 3 d, and 5 d. Patients in groups B, C, and D had significantly lower walking and resting pain than the blank control group, and groups C and D were superior to group B. The results were statistically significant (*P *< 0.05). Nocturnal, flexion pain was significantly lower in patients in groups B, C, and D than in the blank control group (*P *< 0.05). Pain scores were highest at 1 d postoperatively and gradually decreased thereafter (Table 4).

**Table 4:** Comparison of VAS scores.

Categories	Point in time	Score				*P*-value
		Group A	Group B	Group C	Group D	
Walking pain	PRE-OP	55.53 ± 16.94	55.00 ± 13.61	54.60 ± 13.46	54.53 ± 13.43	0.9803
	POD1	65.27 ± 10.62	58.07 ± 13.74	50.47 ± 11.28	50.60 ± 9.72	**<0** **.** **0001**
	POD3	47.40 ± 9.69	42.53 ± 9.45	37.53 ± 9.45	37.73 ± 7.48	**<0** **.** **0001**
	POD5	33.27 ± 6.56	28.33 ± 6.92	24.33 ± 6.92	24.47 ± 5.75	**<0** **.** **0001**
Resting pain	PRE-OP	34.07 ± 7.21	35.13 ± 6.93	35.27 ± 7.13	35.40 ± 7.09	0.722
	POD1	41.60 ± 2.35	35.47 ± 4.95	31.00 ± 4.60	28.00 ± 4.60	**<0** **.** **0001**
	POD3	28.73 ± 3.97	24.87 ± 5.51	21.33 ± 5.34	21.25 ± 5.49	**<0** **.** **0001**
	POD5	23.53 ± 3.41	22.73 ± 6.30	19.73 ± 6.30	18.73 ± 5.91	**<0** **.** **0001**
Nocturnal pain	PRE-OP	42.27 ± 11.64	41.93 ± 8.77	41.63 ± 8.88	41.30 ± 8.38	Total *P*
	POD1	57.33 ± 13.00	52.27 ± 14.42	49.53 ± 14.72	48.27 ± 15.07	**0** **.** **0032**
	POD3	38.80 ± 10.23	32.87 ± 8.89	29.60 ± 8.67	28.73 ± 8.50	**<0** **.** **0001**
	POD5	29.73 ± 5.29	22.67 ± 6.39	19.27 ± 6.17	18.53 ± 5.55	**<0** **.** **0001**
Painful flexion of the knee	PRE-OP	61.13 ± 15.97	60.20 ± 13.53	60.00 ± 12.18	60.27 ± 11.54	0.9687
	POD1	72.40 ± 9.13	65.20 ± 12.12	62.20 ± 12.12	61.93 ± 11.92	**<0** **.** **0001**
	POD3	49.47 ± 11.60	42.67 ± 10.07	39.20 ± 9.89	38.93 ± 9.62	**<0** **.** **0001**
	POD5	35.87 ± 7.96	30.40 ± 8.28	26.93 ± 8.08	25.60 ± 7.06	**<0** **.** **0001**

The bold values represent the statistically significant difference in P-values (i.e., *p* ≤ 0.05).

### 3.4. ERAS indicators

#### 3.4.1. Knee joint circumference

The circumference of the knee joint in the four groups was not significantly different (*P *> 0.05) at 1 d and 5 d postoperatively. At 3 d postoperatively, the mean knee joint circumference in groups B, C, and D were significantly lower than that in the blank control group (*P *< 0.05).

#### 3.4.2. Knee mobility and AKS scores

The degree of motion of knees on the first postoperative day in group C and D was significantly superior to that in the blank control group (*P *< 0.05); at 3 d postoperatively, patients in groups B, C and D were significantly better than the blank control group in this respect (*P *< 0.05); at 5 d postoperatively, patients in groups C and D were significantly better than those in groups A and B in this respect (*P *< 0.05). With respect to knee AKS clinical and functional scores at 3 weeks follow-up, patients in groups B, C, and D were better than the blank control group, and patients in groups C and D were significantly better than patients in groups A and B (*P *< 0.05) (Table 5).

**Table 5:** Comparison of the AKS.

Categories	Preclinical		Functionalities	
	Pre-operative	3 weeks after surgery	Pre-operative	3 weeks after surgery
A	43.67 ± 10.90	75.87 ± 6.72	41.33 ± 12.27	68.00 ± 8.08
B	44.13 ± 11.82	78.33 ± 3.41	41.00 ± 10.32	74.80 ± 7.47
C	43.80 ± 11.85	82.33 ± 3.41	41.13 ± 7.30	78.80 ± 7.47
D	43.60 ± 10.85	82.73 ± 3.41	41.20 ± 6.51	78.40 ± 7.61
*P*	0.9943	**<0**.**0001**	0.9979	**<0**.**0001**

The bold values represent the statistically significant difference in P-values (i.e., *p* ≤ 0.05).

#### 3.4.3. Generalized anxiety scale (GAD-7) scores

Gad-7 scores at discharge in groups B, C, and D were significantly lower than those in the blank control group (*P *< 0.05). Groups C and D were significantly superior to groups A and B (*P *< 0.05), while there was no significant difference (*P *> 0.05) between groups C and D in this respect.

#### 3.4.4. Satisfaction scores

In groups B, C, and D, the satisfaction scores at 1 d, 3 d, and 5 d postoperatively were significantly higher than that in the blank control group (*P *< 0.05). Group D had the highest scores, which were significantly different from those in group B (*P *< 0.05) but not significantly different compared with group C (*P *> 0.05).

#### 3.4.5. Quality of life SF-12 scores

The mean postoperative 3 W physical function, somatic pain, mental health, energy and social function scores in groups B, C, and D were significantly higher than that in the blank control group (*P *< 0.05); patients in group D had higher scores than in group C, but the differences were not statistically significant (*P *> 0.05). Postoperative 3 W physical function, overall health score comparison was not statistically significant (*P *> 0.05), emotional function in group D was significantly higher than that in groups A and B (*P *< 0.05).

### 3.5. Length of stay, hospital costs and perioperative adverse effects

The length of stay and cost of hospitalization in groups B, C, and D were significantly lesser than that in the blank control group (*P *< 0.05); there were no significant differences among the three intervention groups in this respect (*P *> 0.05). There were no significant differences among the four groups in terms of adverse effects such as dizziness or headache (*P *> 0.05). However, the incidence of rebound insomnia and hangover effects were significantly higher in group C than in the other three groups (*P *< 0.05); early awakening was significantly more frequent in groups A and B than in groups C and D, and the difference between the four groups was statistically significant (*P *< 0.05) (Table 6).

**Table 6:** Comparison of perioperative adverse outcomes .

Indicators	A	B	C	D	*P*
Dry mouth	11	10	8	6	0.578
Sweating	5	3	2	2	0.551
Dizzy	9	5	4	3	0.228
Headache	5	3	2	2	0.551
Diarrhea	1	0	1	0	0.569
Gastrointestinal reactions	6	4	2	1	0.161
Bounce-back insomnia	0	0	8	2	**<0** **.** **001**
Sleepiness	0	0	1	0	0.390
Wake up early.	35	20	9	5	**<0** **.** **001**
Hangover effect	0	0	6	1	**0** **.** **002**

The bold values represent the statistically significant difference in P-values (i.e., *p* ≤ 0.05).

## 4. Discussion

### 4.1. Problems with sleep quality in knee arthroplasty

Total knee arthroplasty (TKA) is the primary treatment option for advanced degenerative knee disease or rheumatoid arthritis for the relief of knee pain and improvement of joint deformity and to improve the quality of life of patients (5). Fear of complications associated with TKA and the ambivalence of desperately wanting surgery to improve quality of life can cause preoperative anxiety, which can affect the quality of preoperative sleep of patients. At the same time, TKA is a very traumatic procedure, causing increased pain at night and affecting the quality of sleep (7). In this study, we found that patients generally had poor postoperative sleep, with a 53.3% incidence of sleep disturbance in the blank control group. This is largely consistent with the results of a previous study [5]. The study showed that TKA patients were in a state of high psychological stress and sympathetic excitement after surgery. The consequent elevated concentration of catecholamines in the blood leaves patients in an excited and irritable state, which along with nocturnal knee pain, greatly affects the quality of perioperative sleep after TKA.

### 4.2. Relationship between sleep quality and pain and recovery in knee arthroplasty

Studies have shown a positive correlation between pain and blood levels of inflammatory markers (ESR, CRP, IL-6). Elevated inflammatory factors cause severe postoperative nocturnal pain in patients and greatly affect the quality of postoperative sleep after major orthopedic surgery (7). Postoperative pain necessitates increase in the dosage of NSAIDs and opioids. Since opioids affect the activity of opioid receptors through the same binding sites as endogenous opioid peptides, the consequent increase in opioid concentrations in the patient's blood, and decrease in endogenous enkephalin production, significantly reduces rapid eye movement sleep (REM), increasing the number of awakenings during sleep, decreasing sleep efficiency, and prolonging REM latency (8–11).

Under accelerated rehabilitation hip and knee arthroplasty management, a full range of multimodal analgesic protocols are often required for perioperative patients. Prior to TKA, an over-the-top analgesia program was adopted to reduce perioperative pain. In a study by Mammoto et al., 120 knee arthroplasty patients were prospectively randomized into two groups (test group: celecoxib analgesia; control group: placebo); the results showed that effective postoperative analgesia reduces pain, improves sleep quality, and promotes accelerated recovery (12). In a domestic study, 86 patients with knee joint replacement were randomly divided into two groups (test group: full pain care; control group: conventional analgesia). The results showed that effective preoperative nursing guidance and regular analgesic treatment can effectively reduce postoperative pain, improve sleep quality, and promote accelerated recovery of postoperative joint function. Pain affects sleep, and poor sleep quality in turn affects pain. Both these factors affect early functional exercises and generate anxiety; anxiety in turn affects sleep, and the three factors form a vicious circle (13, 14). Improving perioperative sleep quality can increase the threshold of pain response and reduce daytime fatigue, improve patient satisfaction, and accelerate functional recovery (15, 16).

In the present study, compared with the blank control group, patients in the intervention group (perioperative psycho-behavioral and zolpidem tartrate) showed improved sleep quality in the postoperative period at d1, d3, and d5, and the differences were statistically significant. Among these, the psycho-behavioral intervention + zolpidem tartrate 5 mg group and zolpidem tartrate 10 mg group showed the best results. This may be related to the strong sedative-hypnotic effect of zolpidem tartrate; the mechanism of action is through selective action on γ-aminobutyric acid (GABA) α receptor, after a series of binding and interaction, resulting in opening of chloride channels, causing cell membrane hyperpolarization, and inhibiting neuronal excitation. It does not change the sleep structure, but can prolong the overall sleep time, reduce the time to fall asleep and the number of night awakenings, with no hangover effect on the next day (17). Zolpidem tartrate has several advantages: (1) rebound insomnia is less likely to occur at therapeutic doses; (2) sequential administration of zolpidem with benzodiazepine sleeping pills does not cause rebound; (3) no addictive and withdrawal effects (18). Studies have shown that the perioperative application of oral zolpidem tartrate during joint replacement surgery improves the quality of perioperative sleep, relieves daytime fatigue and drowsiness, accelerates early functional exercise, reduces pain, and promotes early and accelerated recovery (19, 20). Behavioral cognitive therapy showed significant improvement in patients' anxiety and depression indices, and could improve patients' sleep quality (21, 22).

Numerous studies have shown that nighttime sleep deprivation lowers the threshold for walking pain during the day and aggravates pain. Nocturnal pain directly causes sleep disturbance and even sleep deprivation; low-quality sleep in turn increases pain sensitivity, creating a vicious cycle of pain - low quality sleep - aggravated pain - even lower quality sleep, greatly affecting the patient's early rehabilitation exercise (23, 24). In the present study, psycho-behavioral intervention + zolpidem tartrate 5 mg and zolpidem tartrate 10 mg significantly reduced pain in TKA patients at 1st d, 3rd d and 5th d postoperatively during walking, resting, nocturnal and flexed knee states. In a previous study, nocturnal pain was shown to positively correlate with joint swelling and blood concentrations of CRP, ESR, and interleukin 6, which are closely related to local and systemic trauma response after TKA (25). In the present study, blood CRP, ESR, and interleukin 6 levels were not significantly different in the psycho-behavioral intervention + zolpidem tartrate 5 mg and zolpidem tartrate 10 mg groups, but their blood concentrations were greatly reduced compared to the blank control group; in addition, the opioid use was relatively reduced, reducing the effect of opioids on patients' perioperative sleep quality and adjusting the vicious cycle to a virtuous one. The possible mechanisms of action are: psycho-behavioral intervention and zolpidem tartrate can improve the perioperative sleep quality of patients, thus reducing the secretion of inflammatory factors and alleviating the postoperative traumatic inflammatory response; the reduction of inflammatory response can reduce the blood concentration of inflammatory factors in the blood, thus alleviating local and systemic pain, reducing the application of related opioids and realizing the improvement of sleep quality. However, the effect of psycho-behavioral interventions alone is limited; psycho-behavioral interventions + zolpidem tartrate 5 mg can achieve the same effect as zolpidem tartrate 10 mg, reducing the risk of zolpidem tartrate-related complications. Improving sleep quality in TKA patients by means of physical and pharmacological interventions can effectively reduce the inflammatory response, enhance the degree of pain tolerance, reduce the amount of application of sleep-influencing analgesic drugs (opioids), and pain relief in turn improves sleep quality, turning a vicious cycle into a virtuous one and promoting accelerated recovery (26, 27).

### 4.3. Relationship between postoperative sleep quality and postoperative functional recovery, improved quality of life, reduced anxiety and increased satisfaction

Low-quality nocturnal sleep due to postoperative environmental changes and pain results in daytime drowsiness and fatigue, which affects the patient's ability to mobilize early on (28). Robert et al. prospectively included 68 patients who underwent arthroscopic surgery to study the relationship between sleep quality and pain, fatigue index, and analgesic medication dosage. They found that postoperative sleep quality and fatigue index were important factors affecting postoperative pain and early recovery, and that low-quality sleep increased daytime fatigue (29, 30). In this study, postoperative knee mobility of patients in groups C and D was significantly greater than that in the other two groups. This may be related to the fact that combination of pharmacological plus non-pharmacological interventions can significantly improve the quality of perioperative sleep, reducing the patients' daytime sleepiness index. Thus they have more time and energy to perform flexion and extension functional exercises in bed or get out of bed early for walking functional exercises, and increase the confidence in postoperative rehabilitation (25). In this study, the satisfaction of patients in groups C and D was also significantly higher than that in the other two groups at 1 d, 3 d, and 5 d postoperatively; this was inextricably linked to good perioperative functional exercises, early time to get out of bed, and a good sense of experience (26).

A range of factors such as pre-operative concerns about the risks of surgery and anesthesia, unfamiliar surroundings and adaptation to the ward, the extent of post-operative rehabilitation exercises and the speed of recovery induce pre- and post-operative anxiety in patients. Studies have shown that anxiety and pain are independent risk factors for the quality of patients' postoperative sleep; low-quality sleep in turn increases the level of postoperative anxiety and depression. In this study, we also found that both zolpidem tartrate and psycho-behavioral interventions significantly improved patients' anxiety and depression during hospitalization, with zolpidem tartrate 5 mg + psycho-behavioral intervention showing comparable effect as zolpidem tartrate10 mg. This may be related to good perioperative functional exercise, high satisfaction and mild pain levels in patients, leading to early and accelerated recovery, and eliminating some of the factors that contribute to postoperative anxiety.

This study confirmed that psycho-behavioral intervention, psycho-behavioral intervention + oral zolpidem tartrate 5 mg and oral zolpidem tartrate 10 mg can improve the sleep quality-related problems, relieve postoperative pain, inflammatory response, and anxiety, improve patient satisfaction, promote functional recovery, and improve the quality of life of patients undergoing knee replacement surgery. The length of hospital stay and hospitalization cost of the psychopharmacology intervention + zolpidem tartrate 5 mg group were similar to those of the zolpidem tartrate 10 mg group. However, the former group had a lower incidence of perioperative adverse reactions and less severe anxiety; therefore, it was safer and more effective for patients undergoing knee replacement and accelerated recovery. Thus, we recommend psychopharmacology intervention in combination with zolpidem tartrate 5 mg as the optimal intervention for these patients.

## Data Availability

The original contributions presented in the study are included in the article, further inquiries can be directed to the corresponding author.
